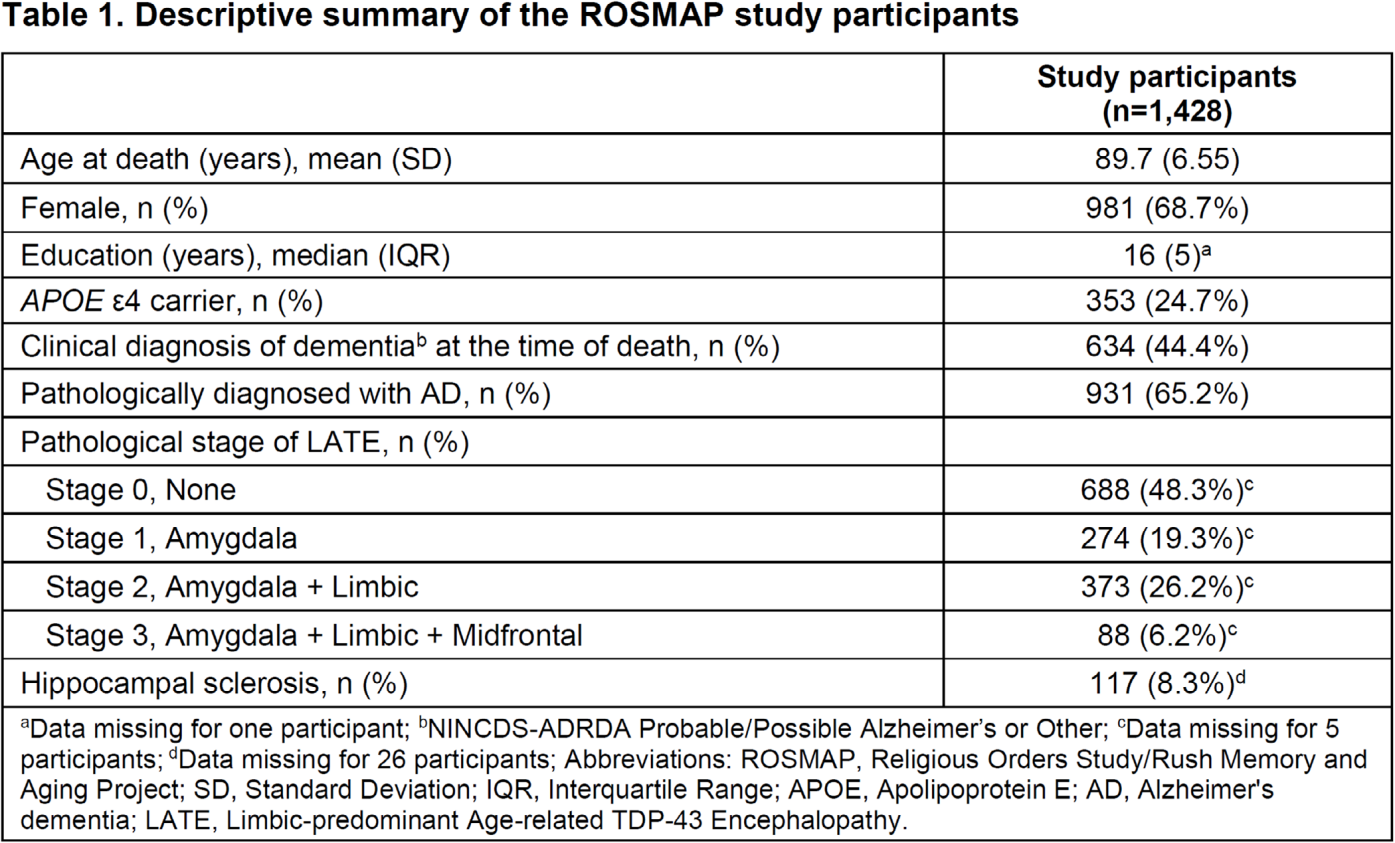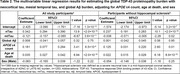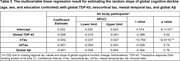# Topographic relationship between tau and TDP‐43 in the combined Alzheimer’s disease and Limbic‐predominant Age‐related TDP‐43 Encephalopathy Neuropathologic Changes

**DOI:** 10.1002/alz.090783

**Published:** 2025-01-03

**Authors:** Jae Won Oh, Sonal Agrawal, Lei Yu, Reisa A Sperling, David A. Bennett, Julie A. Schneider, Hyun‐Sik Yang

**Affiliations:** ^1^ Brigham and Women’s Hospital, Boston, MA USA; ^2^ Harvard Medical School, Boston, MA USA; ^3^ Rush Alzheimer’s Disease Center, Chicago, IL USA; ^4^ Rush University Medical Center, Chicago, IL USA

## Abstract

**Background:**

Alzheimer’s disease (AD) is highly comorbid with Limbic‐predominant age‐related TDP‐43 encephalopathy neuropathological change (LATE‐NC), and the combined AD+LATE‐NC is more common than either pathology alone. However, the topographic relationship between tau and TDP‐43 in AD+LATE‐NC remains unclear.

**Method:**

We analyzed the data from the Religious Orders Study (ROS) and the Rush Memory and Aging Project (MAP) participants. Quantitative amyloid‐β (Aβ) and tau burden were assessed in 8 brain regions using immunohistochemistry; global Aβ burden, mesial temporal tau (mtTau), and neocortical tau (nTau) burdens were calculated. Semiquantitative global TDP‐43 burden was measured with immunohistochemistry in 6 brain regions. We assessed the Aβ–TDP‐43 association with linear regression, adjusting for APOE ε4 count, age at death, and sex. Then, we additionally included nTau and mtTau as predictors in this linear model. We performed mediation analysis to test whether nTau mediates Aβ–TDP‐43 association. We examined the contributions of Aβ, mtTau, nTau, and TDP‐43 on cognitive decline (random slope of change in annual global cognition after controlling age, sex, and education) using a linear model.

**Result:**

We analyzed n = 1,428 deceased ROSMAP participants (Table 1). Global Aβ was positively associated with global TDP‐43 (B = 0.13, p = 2.9×10^‐8^). However, this association was no longer present after including nTau and mtTau in the model; nTau was positively associated with TDP‐43 (B = 0.34, p<2.0×10^‐16^), while mtTau was negatively associated with TDP‐43 (B = ‐0.12, p = 3.6×10^‐11^; Table 2). The negative mtTau–TDP‐43 association was significantly attenuated after excluding participants with hippocampal sclerosis (B = ‐0.038, p = 0.02; Table 2), suggesting that hippocampal sclerosis in advanced LATE‐NC drove this negative association. In a subsequent mediation analysis, nTau completely mediated the Aβ–TDP‐43 association (Proportion mediated: 92.6%, Mediated effect: p<2.2×10^‐16^, Direct effect: p = 0.48). In a multivariable linear model, TDP‐43 and nTau were independently associated with faster cognitive decline, while mtTau and Aβ were not (Table 3).

**Conclusion:**

Our large human neuropathology study supports that, in AD+LATE‐NC, TDP‐43 accumulation might be a downstream consequence of nTau, which fully mediated the Aβ–TDP‐43 association. Both TDP‐43 and nTau exacerbate cognitive decline in AD+LATE. Interventions targeting AD pathophysiology might also delay the progression of comorbid LATE‐NC.